# The Gene Editing of Eukaryotic Translation Initiation Factor Binding Protein 3 and Its Potential Role in Rapid Cold Hardening of Two Invasive Fruit Flies

**DOI:** 10.3390/insects17080844

**Published:** 2026-08-14

**Authors:** Yuning Wang, Rihui Yan, Xianwu Lin, Siya Ma, Lijun Liu, Zhihong Li

**Affiliations:** 1 State Key Laboratory of Agricultural and Forestry Biosecurity, College of Plant Protection, China Agricultural University, Beijing 100193, China; wynzb2333@163.com (Y.W.); ljliu@cau.edu.cn (L.L.); 2 MARA Key Laboratory of Surveillance and Management for Plant Quarantine Pests, College of Plant Protection, China Agricultural University, Beijing 100193, China; 3 Sanya Institute of China Agricultural University, Sanya 572025, China; 4 School of Tropical Agriculture and Forestry, Hainan University, Haikou 570228, China; ryan1@hainanu.edu.cn (R.Y.); linxianw@foxmail.com (X.L.); 5 School of Life and Health Sciences, Hainan University, Haikou 570228, China; masiya1621@outlook.com

**Keywords:** CRISPR-Cas9, Tephitidae, eIF4EBP3, rapid cold hardening, heat shock proteins

## Abstract

*Bactrocera dorsalis* (Hendel) and *Bactrocera correcta* (Bezzi) are two globally concerning quarantine pests within the genus *Bactrocera* (Tephritidae). Both species inflict severe damage on the agricultural industry and international export trade. Their distribution range has expanded due to global warming, posing an increasing threat to fruit production. Heat shock proteins (HSPs), functioning as molecular chaperones, are known to contribute to temperature adaptation in insects. However, the cold adaptation regulatory mechanisms in these two *Bactrocera* species remain unclear. In this study, eIF4EBP3^−/−^ mutations were established by the CRISPR-Cas9 system in both species. The survival rate of the mutations was significantly reduced with cold treatments, and qRT-PCR analysis indicated that eIF4EBP3 regulates the expression of several downstream heat shock proteins, suggesting that it may be involved in rapid cold hardening (RCH) adaptability in both fruit fly species. In addition, this work represents the first application of the CRISPR-Cas9 system in *B. correcta*, which provides methods to further studies on this species. Our results provide insights into the molecular mechanisms underlying rapid cold hardening adaptation in *B. dorsalis* and *B. correcta* and a potential molecular marker for monitoring cold tolerance in field populations which could guide the development of novel control strategies that target the RCH adaptation pathways.

## Simple Summary

*Bactrocera dorsalis* (Hendel) and *Bactrocera correcta* (Bezzi) are two invasive tephritid pests that keep spreading due to global warming in recent years, causing great damage to fruits. Understanding the mechanisms underlying rapid cold hardening is essential for predicting and preventing their colonization of new areas; however, these mechanisms have been rarely investigated. In this study, we knocked out eukaryotic translation initiation factor binding protein 3, which is upstream of some heat shock proteins and is important in rapid cold hardening regulation. The survival rate of the mutations was significantly reduced after cold treatments, and qRT-PCR analysis indicated that the expression levels of several downstream heat shock proteins were significantly changed, suggesting that eIF4EBP3 may be involved in rapid cold hardening (RCH) adaptability in both fruit fly species. This work represents the first application of the CRISPR-Cas9 system in *B. correcta*, which helps further studies on this species. Our results provided information on the molecular mechanisms underlying rapid cold hardening adaptation in *B. dorsalis and B. correcta* and a potential molecular marker for monitoring cold tolerance in field populations, and they could guide the development of novel control strategies that target the RCH adaptation pathways and help predict and prevent damage in the fruit industry.

## 1. Introduction

*Bactrocera dorsalis* (Hendel) and *Bactrocera correcta* (Bezzi) are two invasive tephritid pests of global concern, belonging to the genus *Bactrocera* in the family Tephritidae, causing great damage to the agricultural industry and export trade [1,2]. Despite existing control measures—including sex pheromone traps and chemical pesticides—their expansion and dispersal remain unstoppable [3]. Currently, *B. dorsalis* shows a clear northward expansion trend across 75 countries [4], whereas *B. correcta* remains largely confined to north and northeast Asia, although the two species are morphological similar and share many hosts [5,6]. We hypothesize that their differing geographical ranges may be attributed to variation in overwintering and colonization capacity—traits closely linked to their respective resistance and molecular mechanism under rapid cold hardening (RCH).

Temperature affects the geographic distribution and dispersal of insects by limiting their physiological, biochemical, and other activities [7]. Insects are able to enhance their ability to resist damage from extremely low temperatures after experiencing a period of mild low temperatures higher than the extremely low temperatures, a phenomenon known as rapid cold hardening [8]. During RCH, insects undergo a series of behavioral and physiological adjustments, including the accumulation of cryoprotectants such as polyols within their bodies. These changes significantly enhance the insect’s ability to resist low-temperature injury [9]. In terms of the mechanism of RCH adaptation in *B. dorsalis*, *Klebsiella michiganensis* BD177 enhances the adaptation to RCH by stimulating arginine and proline metabolism [10]. One investigation revealed alterations in the supercooling point of *B. dorsalis* after rapid cold hardening (RCH) and found that the supercooling point of *B. dorsalis* decreased significantly after RCH adaptation, and the content of protein, glycerol, and alginate increased, while the water content decreased [11]. In terms of molecular mechanisms, it has been shown that *Hsp23*, *Hsp27*, *Hsp68*, and *Hsp70* play regulatory roles in low-temperature plasticity in *B. dorsalis* and *B. correcta* [12]. However, the molecular mechanism underlying the RCH adaptation of these two species of *Bactrocera* remains unclear.

Clustered Regularly Interspaced Short Palindromic Repeats (CRISPRs) were originally a defense system for adaptive immunity present in bacteria and archaea. The CRISPR-Cas9 system, which uses RNA to direct Cas9 endonuclease, has been used in the development of genome-editing technology [13]. The technology is simple and reusable, and it can easily examine multi-gene interactions and functions in cells or animals [14]. CRISPR-Cas9 gene-editing technology is rapidly evolving. Yuan et al. [15] systematically summarized the protocol of CRISPR/Cas9 gene editing in *B. dorsalis*, which consists of the design and synthesis of guide RNAs, embryo collection, embryo microinjection, embryo incubation and rearing, and mutant detection and screening. Currently, the application of gene editing in *B. dorsalis* mainly focuses on the study of eye color, reproductive ability and odor receptor function [16,17,18]. However, there is no report on the application of CRISPR-Cas9 gene editing in guava fruit fly.

Eukaryotic translation initiation factor (eIF) regulates the translation process, which has been reported to play an important role in the regulation of protein translation rates in cells, and it has been demonstrated that eIF4EBP is tightly associated with the cellular cold stress response and is upstream of the HSP genes in the longevity regulating pathway which belongs to KEGG pathways [19,20]. Based on our previous study, the RCH adaptation of *B. dorsalis* and *B. correcta* may be regulated by several HSPs, and transcriptome results indicate that the expression level of *eIF4EBP3* in two species was extremely up-regulated [12]. So, we hypothesize that eIF4EBP3 may be involved in the RCH adaptation process in *B. dorsalis* and *B. correcta* by regulating the expression of heat stress protein genes.

In this study, the CRISPR-Cas9 system was used in two *Bactrocera* species to determine whether eIF4EBP3 regulates their RCH adaptation. In addition, we found several heat shock protein genes that may be downstream of eIF4EBP3 and help fruit flies withstand cold temperatures. This is the first study to successfully use gene engineering in *B. correcta* and offers more information on the RCH adaptation mechanisms of two *Bactrocera* species.

## 2. Materials and Methods

### 2.1. Fruit Fly Rearing

The *B. dorsalis* population was collected in Guangzhou (23.40° N, 113.22° E), Guangdong province, China, while the *B. correcta* population was collected in Yuanjiang (23.60° N, 102° E), Yunnan province, China. The fruit flies were maintained in the laboratory at 25 °C with 70% relative humidity under a 14:10 (L:D) period. The adults were provided with an adult diet containing sucrose and peptone (3:1 by weight) and water. The eggs were collected in perforated paper cups containing diluted mango juice for further embryo treatment and injection.

### 2.2. Synthesis of Cas9 mRNA and sgRNA

The pTD1-T7-Cas9 plasmid was linearized using NotI-HF (NEB, Beijing, China) and FastAP Thermosensitive Alkaline Phosphatase (Thermo Scientific, Waltham, MA, USA). The same volume of nuclease-free water was added into the linearized plasmid, and the whole volume was calculated. The same volume of the phenol:chloroform:isoamyl alcohol (25:24:1) mixture (PCI) was added, and the mixture was centrifuged at 4 °C and 13,000× *g* for 10 min. The supernatant was transferred to a new EP tube, and 10% by volume of 3M NaAc was added. After inverting and mixing, 600 μL of anhydrous ethanol was added. It was then placed at −80 °C for more than 20 min to allow precipitation to occur. The mRNA was produced by the mMESSAGE mMACHINE^®^T7 Kit (Life Technologies, Shanghai, China) from 0.5 μg of linearized DNA. The Cas9-encoding mRNA was purified (same as purification of the linearized plasmid) and resuspended in RNase-free water.

The eIF4EBP3 sequences of both species were retrieved from our transcriptome data. Potential target sites within the exon regions of the open reading frame were selected based on the GG/CN18NGG principle using CRISPOR v5.2 (Figures S1 and S2 and Table S1) [21]. Three pairs of primers were designed for each species to knock out eIF4EBP3. Each sgRNA was encoded by a pair of primers: a forward primer containing the T7 promoter and the target-specific sequence and a common reverse primer encoding the sgRNA scaffold (Table S1). The primer pairs were amplified via PCR using PrimeSTAR^®^ HS DNA Polymerase (TaKaRa, Beijing, China), with each reaction performed in triplicate (50 μL per tube). PCR products were purified with the TIANgel Purification Kit (Tiangen, Beijing, China) and cloned into the linearized pJET1.2/blunt vector (Thermo Scientific, Shanghai, China). DNA templates for in vitro transcription were amplified from the purified plasmids using KOD-Plus-DNA polymerase (TOYOBO, Shanghai, China) with primers in Table S1. Finally, sgRNAs were synthesized using the MEGAscript T7 Kit (Thermo Scientific, Shanghai, China) according to the manufacturer’s protocol.

### 2.3. Embryo Injection

Embryo injection was performed at 18 °C in order to slow the polarization of eggs. Newly laid eggs were collected by putting perforated paper cups containing diluted mango juice in cages of fruit flies for 15 min. Then eggs were placed on glass slides such that their heads were arranged in one direction. The glass slides with eggs were placed on a dehumidifier for 10 min before microinjection. A drop of halogenated hydrocarbons [halocarbon oils (Sigma, Shanghai, China) 700:27 = 1:1] was covered placed on the eggs. Purified Cas9 mRNA and sgRNA were combined in the injection buffer (5 mM KCl, 0.1 mM sodium phosphate, pH 7.4) at final concentrations of 0.5 and 0.25 μg·μL^−1^, respectively [18]. Pre-blastoderm embryos were co-injected at the posterior end using a Femtojet 4i microinjector (Eppendorf, Shanghai, China) under a Nikon SMZ18 stereomicroscope (Shanghai, China). Following injection, embryos were gently wiped, transferred to an artificial climate incubator at 25 °C, and maintained until larval hatching. The larvae were subsequently reared on an artificial diet under the same conditions as the wild-type (WT) flies. For each species, three distinct sgRNAs targeting different sites within the eIF4EBP3 gene were designed (Table S1). A total of 250 embryos per experimental group were subjected to microinjection of the Cas9 mRNA/sgRNA mixture, whereas control (WT) embryos were injected with sterile water.

### 2.4. Detection of Mutations and Crosses

Genomic DNA was isolated from a single leg of each G0 fly using the TIANamp Micro DNA Kit (Tiangen, Beijing, China) and subsequently employed for targeted mutation detection. PCR was performed using genomic DNA, 2× Lightning Taq PCR MasterMix (GeneBetter, Beijing, China) and primers designed to amplify an approximately 1000 bp PCR product (500 bp upstream and downstream of the target site). PCR products were inserted into the pGEM-T easy vector (Promega, Beijing, China) for sequencing, which was performed by Beijing Tsingke Biotech Co., Ltd., China. Within each developmental stage, pairwise comparisons among all groups (WT, -1, -2, -3) were performed using two-sample proportion Z-tests in SPSS 25.0.0.0. Heterozygous G0 individuals (eIF4EBP3^−/+^) with the confirmed mutation were back-crossed to WT flies, while heterozygous G1 individuals (eIF4EBP3^−/+^) were self-fertilized to observe the homozygous (eIF4EBP3^−/−^) mutations in G2 individuals. The eIF4EBP3^−/−^ population was then established by self-fertilization of homozygous (eIF4EBP3^−/−^) mutations in G2. To ensure eIF4EBP3 was knocked out, qRT-PCR was carried out to confirm the absence of eIF4EBP3 mRNA.

### 2.5. Temperature Treatment and mRNA Detection

After the homozygous eIF4EBP3^−/−^ population was established, eggs were collected during their fertile period. Temperature treatment was performed after 7 days. Each group had 5 replicates of 40 3rd-early-instar larvae per tube with holes on their lids. The treatment was performed as follows: (a) Larvae were subjected to 4 h of RCH (9 °C for *B. dorsalis* and 5 °C for *B. correcta*) and 1 h of recovery. Then, 10 larvae per tube were collected for mRNA detection. (b) Larvae were subjected to extremely low temperatures for 1 h (−7.8 °C for *B. dorsalis* and −8.3 °C for *B. correcta*) and 4 h of recovery [12]. After that, the survival rate was calculated by touching the larvae with tweezers; if no reaction was observed after touching, the larvae would be counted as dead. RNA extraction was performed using an Tiangen RNA extraction kit. Following reverse transcription (PrimeScript RT Reagent Kit with gDNA Eraser; TaKaRa, Beijing, China), gene expression was quantified by qRT-PCR on an ABI QuantStudio 6 Flex instrument (Thermo Fisher, Shanghai, China) using TaKaRa SYBR Premix Ex Taq II. Three technical replicates were included per sample, and *18S* was used as the internal reference. Expression levels were calculated using the 2^−ΔΔCT^ method, and statistical comparisons were performed with two-way ANOVA in SPSS 25.0.0.0.

## 3. Results

### 3.1. Targeted Mutagenesis of eIF4EBP3 in Two Species

**Figure 1:**
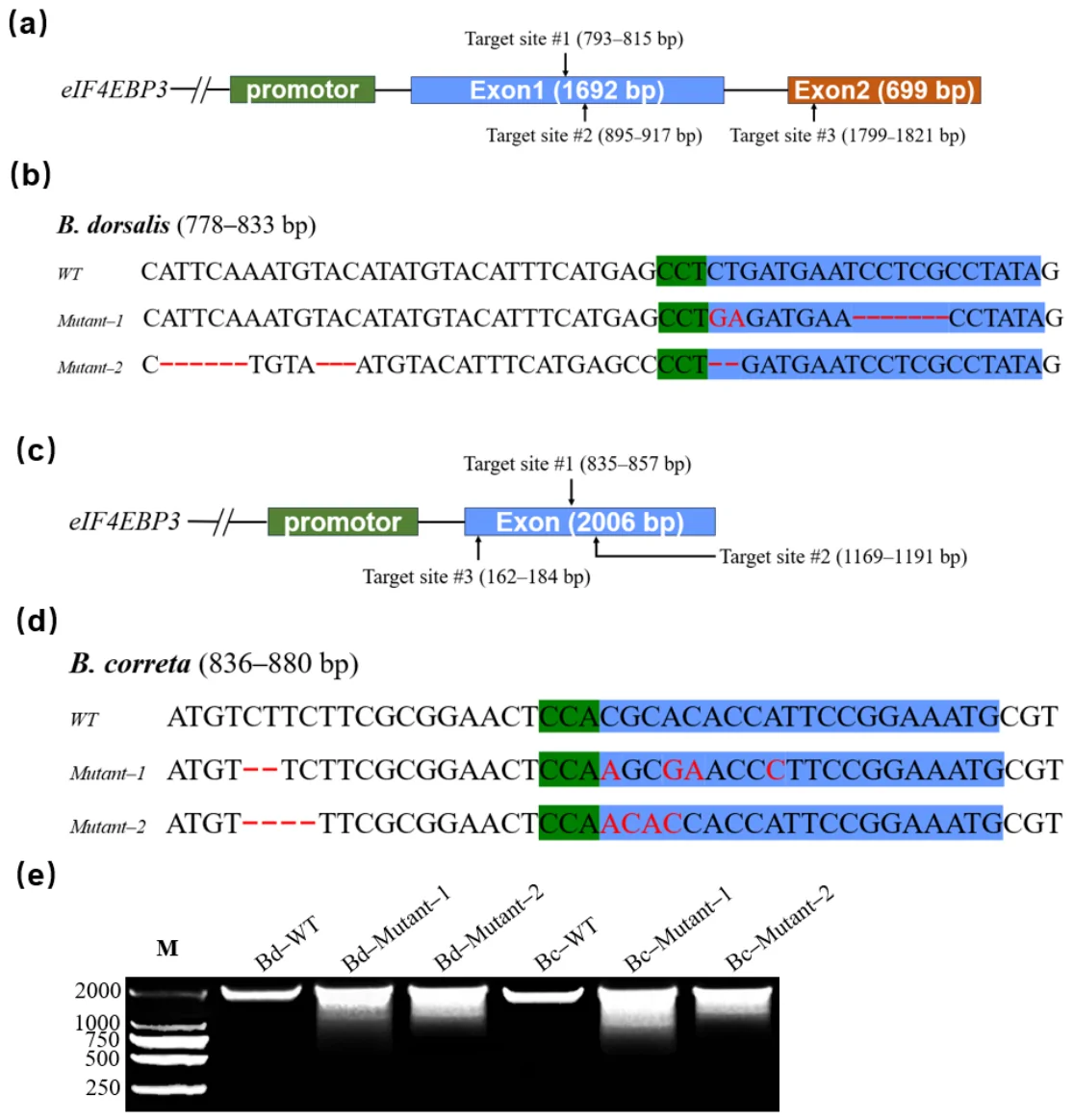
eIF4EBP3 target site and sequence of homozygous mutations in *Bactrocera dorsalis* and *Bactrocera correcta.* (**a**) Target site of eIF4EBP3 in *B. dorsalis*; (**b**) sequences of the WT and mutations in *B. dorsalis*. (**c**) Target site of eIF4EBP3 in *B. correcta*; (**d**) sequences of the WT and mutations in *B. correcta*. (**e**) Agarose gel electrophoresis results of the wild-type group and mutations of two species. The codons in blue means target sequence, and codons in green means PAM sequence. #: Number; M: marker; WT: wild type; Bd: *B. dorsalis*. Bc: *B. correcta*.

**Figure 2:**
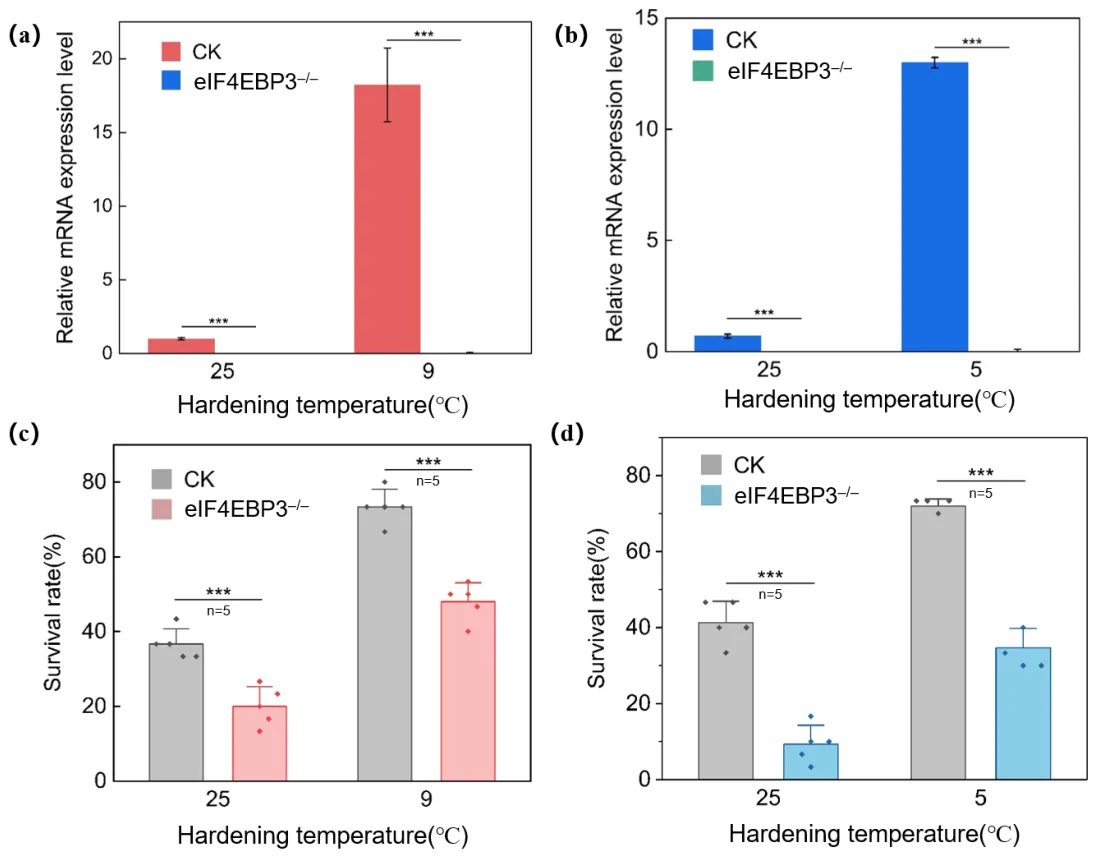
Expression level and survival rate after knocking out eIF4EBP3 in *Bactrocera dorsalis* and *Bactrocera correcta*. Note: (**a**) Expression level of *eIF4EBP3* after editing the eIF4EBP3 gene of *B. dorsalis*. (**b**) Expression level of *eIF4EBP3* after editing the eIF4EBP3 gene of *B. correcta*. (**c**) Survival rate at extremely low temperatures after knocking out the eIF4EBP3 gene of *B. dorsalis*. (**d**) Survival rate at extremely low temperatures after knocking out the eIF4EBP3 gene of *B. correcta*. The expression level of the CK group was set to “1”. “***” means *p* < 0.001, as determined by the two-way ANOVA test. Each bar indicates the SEM of each group.

The target sites of eIF4EBP3 and sequence changes in the homozygous mutations of the two species are shown in Figure 1. The numbers of injected embryos, hatched larvae, pupae, emerged individuals and mutations of two species are shown in Table 1 and Table 2. Compared to the wild type, Bd-eIF4EBP3-1 and Bc-eIF4EBP3-1 not only significantly reduced survival rates but also efficiently induced targeted mutations; in contrast, Bd/Bc-eIF4EBP3-2 and -3 caused significant developmental lethality but no mutation was detected. Two types of eIF4EBP3^−/−^ mutations were detected in *B. dorsalis*, and the sequence changes exhibited base deletions at positions 896 to 899 or positions 886 to 891 and base substitutions including PAM sequences at position 900 or 902, which caused the changes in eIF4EBP3’s amino acid sequence (Figure 1a,b). In the two eIF4EBP3^−/−^ mutations detected in the guava fruit fly, the substitution of the base at position 866 in m1 resulted in the substitution of the codon encoding histidine to leucine, and the m2 mutant had a base deletion (Figure 1c,d). The smearing bands in the agarose gel confirmed the presence of the mutation (Figure 1e). The qRT-PCR test confirmed the almost complete absence of *eIF4EBP3* mRNA in eIF4EBP3^−/−^ flies (Figure 2a,b).

### 3.2. RCH Adaptation Ability After Knocking out eIF4EBP3

After the establishment of the eIF4EBP3^−/−^ mutant in two species, we measured the survival rate under extremely low temperatures after RCH. Compared with the wild type, both mutant strains exhibited a marked reduction in survival rate. The survival rate of *B. dorsalis* at extremely low temperatures following hardening at 9 °C decreased from 36.67 ± 3.65% to 20 ± 2.67%, and that of *B. correcta* at extremely low temperatures after hardening at 5 °C decreased from 41.3 ± 4.99% to 10 ± 3.65% (Figure 2c,d), which suggests that eIF4EBP3 may play a role in the RCH adaptation mechanism of two *Bactrocera* species.

### 3.3. Heat Shock Protein Genes’ Expression After Knocking out eIF4EBP3

**Figure 3:**
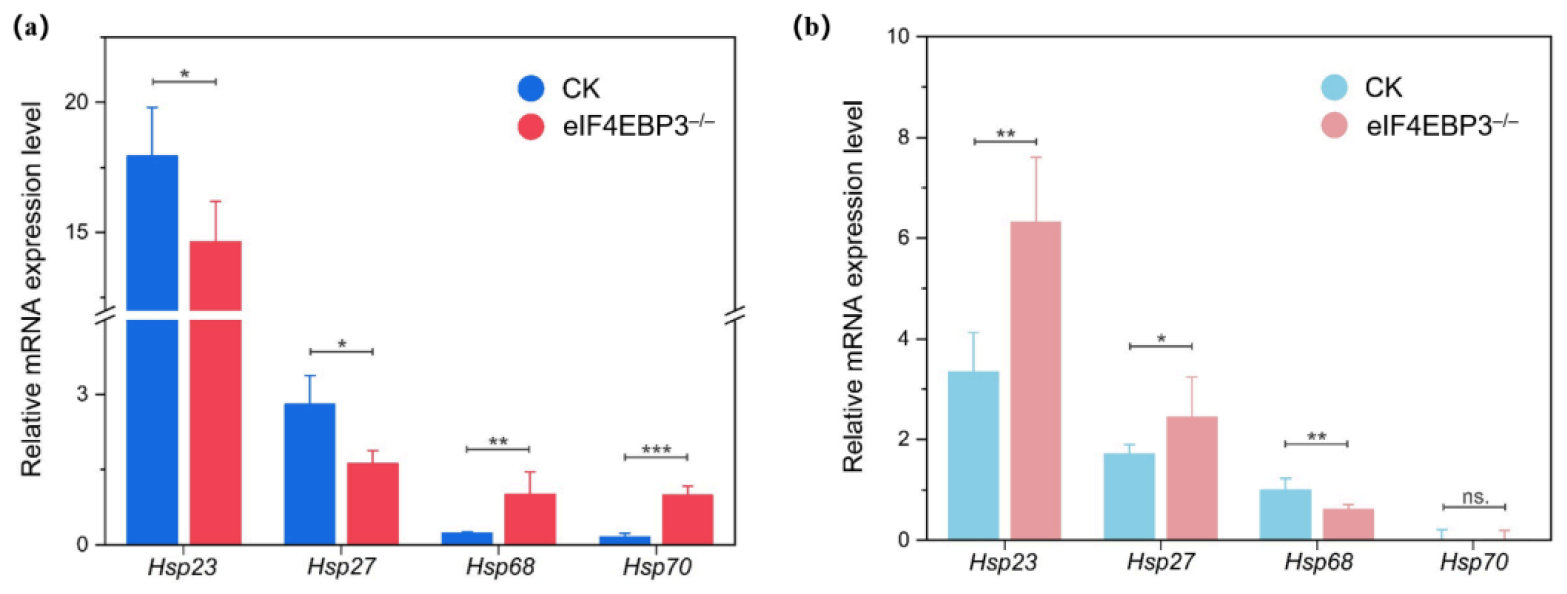
Expression levels of *Hsp23*, *Hsp27*, *Hsp68* and *Hsp70* after editing eIF4EBP3 in *Bactrocera dorsalis* and *Bactrocera correcta*. Note: (**a**) Expression levels of *Hsp23*, *Hsp27*, *Hsp68* and *Hsp70* in *B. dorsalis*. The expression level of *Hsp68* in the CK group was set to “1”. (**b**) Expression levels of *Hsp23*, *Hsp27*, *Hsp68* and *Hsp70* in *B. correcta.* The expression level of *Hsp70* in the eIF4EBP3^−/−^ group was set to “1”. “*” means *p* < 0.05, “**” means *p* < 0.01, “***” means *p* < 0.001, and “ns.” means *p* > 0.05, as determined by the two-way ANOVA test. Each bar indicates the SEM of each group.

For the purpose of understand how eIF4EBP3 regulated RCH adaptation in *B. dorsalis* and *B. correcta*, qRT-PCR was performed to test the expression of four heat shock protein genes that may regulate the adaptation of RCH in two species. In *B. dorsalis* larvae with eIF4EBP3^−/−^ mutations, the expression levels of *Hsp23* and *Hsp27* were significantly decreased (by 18% and 42%, respectively), and the expression of *Hsp68* and *Hsp70* increased 3.99-fold and 4.95-fold, respectively (Figure 3a). In the guava fruit flies with eIF4EBP3^−/−^ mutations, the expression of *Hsp23* and *Hsp27* increased 1.89-fold and 1.42-fold, and the expression of *Hsp68* decreased by 38%, while the expression of *Hsp70* was not significantly different and nearly undetectable (Figure 3b). This result suggests that eIF4EBP3 may regulate the expression of heat shock proteins in two *Bactrocera* species.

## 4. Discussion

As CRISPR-Cas9 technology has been available for a few years, it has been applied in fruit flies, including *Ceratitis capitata*, *Bactrocera tryoni*, etc. [22,23]. To date, gene editing is still based on embryo injection, which is hard for smaller insects. The first application of the CRISPR/Cas9 system in *B. dorsalis* was reported in 2018 [18]; since then some methods of egg injection and mutant detection in *B. dorsalis* have been published, which offered effective protocols in the gene editing of *B. dorsalis* [24]. However, it remains challenging to inject these small embryos after pre-injection treatment. In this study, newly laid eggs were microinjected directly after drying them for 10 min and covering them with halogenated hydrocarbons, which saved time and reduced the difficulty of injections. Three sgRNAs were designed for both species to knock out eIF4EBP3, and only one sgRNA succeeded. This was possibly because of one-target-site gene editing, as well as the lack of high-quality genome data of *B. correcta*, as the SNPs may affect the efficiency of genome engineering. This study explores embryo microinjection without complex treatments, and it is the first report of genome engineering in *B. correcta* using the CRISPR-Cas9 system, which may be useful for gene editing in fruit flies or other small species.

Eukaryotic translation initiation factors regulate the initiation of the translation process [25]. eIF4EBP2 is upstream of heat shock proteins in the longevity regulating pathways of flies, while eIF4EBP3 has mostly been reported in cancer studies of humans or chemical structures and has not been reported in insects or the cold hardening process [26]. In this study, we tested whether eIF4EBP3 contributed to the adaptation of RCH of two species. After gene editing of eIF4EBP3 in *B. dorsalis* and *B. correcta*, the survival rate following cold treatments was significantly down-regulated, and the expression levels of HSPs also changed significantly. We suppose that eIF4EBP3, as a transcription regulatory element, probably contributes to the regulation of both species under RCH by regulating the expression of HSPs.

According to the gene expression results, the expression patterns of heat shock protein genes related to RCH adaptation in two types of fruit flies are different. In *B. dorsalis* eIF4EBP3^−/−^ mutants, the expression levels of four HSPs were totally different from those of *B. correcta* eIF4EBP3^−/−^ mutants. We assume that *B. dorsalis* might resist low temperatures by regulating the expression of small heat shock proteins, while *B. correcta* might resist low temperatures by regulating the expression of large-molecular-weight heat shock proteins. The two species of fruit flies use different molecular regulatory pathways to cope with cold, and this need further studies to confirm. In addition, after gene editing of eIF4EBP3, the expression level of *Hsp70*, which was reported to play a role in temperature adaptation, was upregulated in *B. dorsalis*, but it could hardly be detected in *B. correcta* [12]. These differential expression patterns may partly explain why *B. dorsalis* exhibits greater cold resilience, a wider geographical distribution and rapid northward expansion compared to *B. correcta* [27].

## 5. Conclusions

In this study, we knocked out Eukaryotic translation initiation factor binding protein 3 in *B. dorsalis* and *B. correcta* and found that it affects rapid cold hardening adaptation by regulating the expression of heat shock proteins. Our results provide a potential molecular marker for monitoring cold tolerance in field populations and could guide the development of novel control strategies that target the RCH pathway. Additionally, this study first applied gene editing in *B. correcta*, which supports further studies on this species.

## Figures and Tables

**Table 1:** Survival rate and mutagenesis after microinjection of *Bactrocera dorsalis* (Bd).

**Group**	**Injected Embryos**	**Hatched Larvae** **(Survived/Dead)**	**Pupae** **(Survived/Dead)**	**Emerged Individuals** **(Survived/Dead)**	**Mutant Percentage (Emerged Adults)**
Bd-eIF4EBP3-1	250	36.8% (92/250) ^b^	56.5% (52/92) ^b^	38.5% (20/52) ^b^	25.0% (5/20) ^a^
Bd-eIF4EBP3-2	250	32.8% (82/250) ^b^	52.4% (43/82) ^b^	41.9% (18/43) ^b^	0 ^b^
Bd-eIF4EBP3-3	250	16.8% (42/250) ^c^	45.2% (19/42) ^b^	57.9% (11/19) ^b^	0 ^b^
Bd-WT	250	60.4% (151/250) ^a^	72.2% (109/151) ^a^	83.5% (91/109) ^a^	0 ^b^

Note: Different superscript lowercase letters within the same column indicate significant differences at *p* < 0.05 (two-sample proportion Z-test).

**Table 2:** Survival rate and mutagenesis after microinjection of *Bactrocera correcta* (Bc).

**Group**	**Injected Embryos**	**Hatched Larvae** **(Survived/Dead)**	**Pupae** **(Survived/Dead)**	**Emerged Individuals** **(Survived/Dead)**	**Mutant Percentage (Emerged Adults)**
Bc-eIF4EBP3-1	250	22.4% (56/250) ^b^	53.6% (30/56) ^b^	56.7% (17/30) ^b^	23.5% (4/17) ^a^
Bc-eIF4EBP3-2	250	21.2% (53/250) ^b^	54.7% (29/53) ^b^	41.4% (12/29) ^b^	0 ^b^
Bc-eIF4EBP3-3	250	22.4% (56/250) ^b^	53.6% (30/56) ^b^	53.3% (16/30) ^b^	0 ^b^
Bc-WT	250	65.2% (163/250) ^a^	71.2% (116/163) ^a^	76.7% (89/116) ^a^	0 ^b^

Note: Different superscript lowercase letters within the same column indicate significant differences at *p* < 0.05 (two-sample proportion Z-test).

## Data Availability

The genes in this study were selected through transcriptome data. The raw data are available upon request due to their relevance to our next manuscript which is related to eukaryotic translation initiation factors’ functions and interactions.

## Supplementary Materials

The following supporting information can be downloaded at https://www.mdpi.com/article/10.3390/insects17080844/s1, Table S1: Primers used in this study. Table S2: Sequence information of heat shock proteins used in this study. Figure S1: Parameter settings for designing sgRNA in CRISPOR. Figure S2: The results of designing sgRNA. (For example).
