# Supplementary material for: The Gene Editing of Eukaryotic Translation Initiation Factor Binding Protein 3 and Its Potential Role in Rapid Cold Hardening of Two Invasive Fruit Flies
Source: Insects. 2026 Aug 14;17(8):844. doi: 10.3390/insects17080844 (PMC13513019; doi:10.3390/insects17080844)
Supplement: Supplementary file 1 [file insects-17-00844-s001.zip › insects-4432893-supplymentary.pdf]

Table S1 Primers used in this study.

Name	Sequence	Usage
Kn4E-bd1	TAATACGACTCACTATAGGTATAGGCGA GGATTCATCAGAGGTTTTAGAGCTAGAA ATAGCAAGTTAAAATAAGGCTAGTCC	Synthesize sgRNA
Kn4E-bd2	TAATACGACTCACTATAGGTCATGTATT ATAGTCCCGCAAGGTTTTAGAGCTAGAA ATAGCAAGTTAAAATAAGGCTAGTCC	Synthesize sgRNA
Kn4E-bd3	TAATACGACTCACTATAGGGTGTGCGTG GAGTTCCGCGAAGGTTTTAGAGCTAGA AATAGCAAGTTAAAATAAGGCTAGTCC	Synthesize sgRNA
Kn4E-bc1	TAATACGACTCACTATAGGCATTTCCGG AATGGTGTGCGTGGTTTTAGAGCTAGAA ATAGCAAGTTAAAATAAGGCTAGTCC	Synthesize sgRNA
Kn4E-bc2	TAATACGACTCACTATAGGGGCGTAGC GCAACATCGTAGTGGTTTTAGAGCTAGA AATAGCAAGTTAAAATAAGGCTAGTCC	Synthesize sgRNA
Kn4E-bc3	TAATACGACTCACTATAGGTAAAGCCG AAGTCGTCGTCGCGGTTTTAGAGCTAGA AATAGCAAGTTAAAATAAGGCTAGTCC	Synthesize sgRNA
Kn4E-R	AAAAGCACCGACTCGGTGCCACTTTTTC AAGTTGATAACGGACTAGCCTTATTTTA	Synthesize sgRNA

	ACTTGCTATTTCTAGCTCTAAAA	
PJET-F500	TAATGTGAGTTAGCTCACTCATTAG	Synthesize sgRNA
sgRNA-R20	AAAAGCACCGACTCGGTGCC	Synthesize sgRNA
DT4E-BD-F	AGCTTATCTACGAACGCGCA	Check mutation
DT4E-BD-R	GGTCAATATGACCGAAAGTTGAT	Check mutation
DT4E-BC-F	AGTCTAGCAAACAGCGAGGT	Check mutation
DT4E-BC-R	CGGATGTGGATGACTTATAACGG	Check mutation

Notes: The target sequences are marked in red.

Table S2 Sequence information of heat shock proteins used in this study.

Name	GenBank access number/status
Hsp23-bd	PV806176.1
Hsp27-bd	XM_011199814.3
Hsp68-bd	XM_049448536
Hsp70-bd	XM_049446617.1
Hsp23-bc	Submitting to NCBI
Hsp27-bc	Submitting to NCBI
Hsp68-bc	Submitting to NCBI
Hsp70-bc	JX961639.1

Note: “bd” is for *Bactrocera dorsalis* while “bc” for *Bactrocera correcta*.

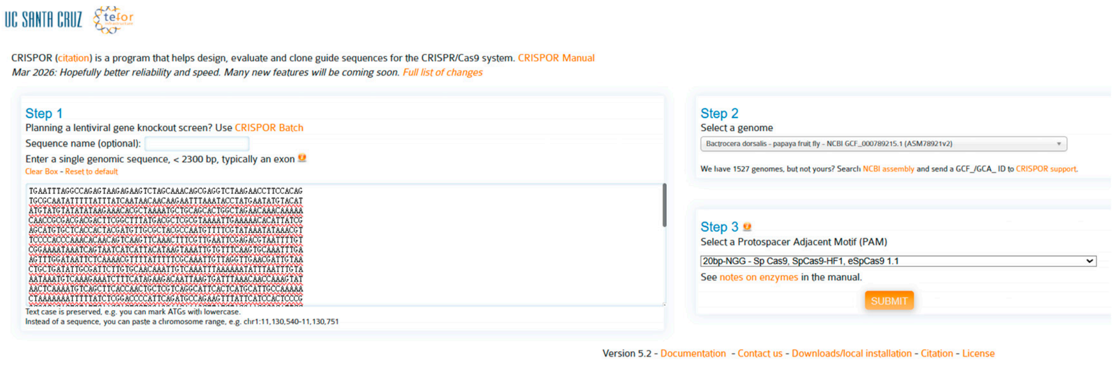

Figure S1 Parameter settings of designing sgRNA in CRISPOR.

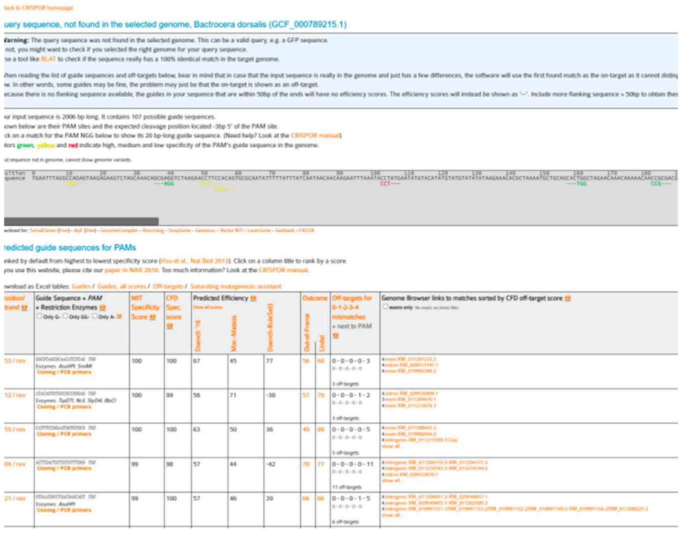

Figure S2 The results of designing sgRNA. (For example)
